# Date Fruit (*Phoenix dactylifera* L.) Cultivar Extracts: Nanoparticle Synthesis, Antimicrobial and Antioxidant Activities

**DOI:** 10.3390/molecules27165165

**Published:** 2022-08-13

**Authors:** Abdulghani Ashraf Halabi, Bassma H. Elwakil, Mohamed Hagar, Zakia A. Olama

**Affiliations:** 1 Department of Botany & Microbiology, Faculty of Science, Alexandria University, Alexandria 21568, Egypt; 2 Department of Medical Laboratory Technology, Faculty of Applied Health Sciences Technology, Pharos University in Alexandria, Alexandria 21321, Egypt; 3 Department of Chemistry, Faculty of Science, Alexandria University, Alexandria 21568, Egypt

**Keywords:** *Phoenix dactylifera*, antimicrobial, antioxidant, nano-fresh fruit of ”Hayany” date extract/amikacin

## Abstract

The pharmaceutical research sector’s inability to produce new drugs has made it difficult to keep up with the rate at which microbial resistance is developing. Recently, nanotechnology and its combinations with natural products have been the saviors against multidrug resistant bacteria. In the present investigation, different Egyptian and Saudi date cultivars were extracted and then phytochemically analyzed and tested for possible antimicrobial activities against multidrug resistant (MDR) microbes. The results revealed that extract of the flesh of fresh “Hayany” fruit (Egyptian date) showed the highest antimicrobial activity, with high levels of phenolic, flavonoid, and tannin concentrations (538.578 µg/mL, 28.481 µg/mL, and 20.888 µg/mL, respectively) and high scavenging activity, with an IC50 reaching 10.16 µg/mL. The highest synergistic activity was found between fresh “Hayany” fruit extract and amikacin. Novel nano-fresh fruit of “Hayany” date extract was synthesized using a ball-milling technique. The vesicle size was 21.6 nm, while the PDI and zeta potential were 0.32 and +38.4 mV, respectively. The inhibition zone diameters of nano-fresh fruit of “Hayany” date extract/amikacin reached 38 mm and 34 mm, with complete microbial eradication after 9 h and 6 h, against *Candida albicans* and *Staphylococcus aureus*, respectively. In conclusion, date fruit extract could be used as a candidate bioactive compound in the fight against infectious diseases.

## 1. Introduction

Multidrug resistant (MDR) bacteria pose a serious hazard to the entire world and have lessened the treatment options against infectious diseases. There is a need for more effective antimicrobial agents against MDR infections even though traditional antimicrobial drugs are fairly effective against some communicable diseases [1]. There is growing urgency for the invention of plant-based natural pharmaceutical products for the treatment and prevention of pathogenic diseases. Herbal medicines and phytochemicals have been employed for their promising antibacterial action since the prehistoric period. Moreover, the application of antimicrobial agent/phytochemical combinations in pharmaceutical industry can weaken the resistance mechanisms of MDR bacteria and enable the antibiotic to regain its effectiveness against multidrug resistant microorganisms [1,2]. These phytochemicals possess antimicrobial activity through inhibiting the efflux pump, degrading some resistant enzymes, etc. There have been several reports of herbal extracts, essential oils, and isolated pure chemicals acting in combination with some already available antibiotics, antifungals, and chemotherapeutics to increase the potency of these medications. The sensitivity patterns of numerous microorganisms towards natural/antimicrobial combinations showed considerable declines in the minimum inhibitory concentrations (MICs) [1].

An abundant source of bioactive substances with a variety of health advantages can be found in fruits and vegetables [2]. Frequently, nutritional antioxidants such as polyphenols, carotenoids, ascorbic acid, and tocopherols have been implicated. In recent studies, there has been increased interest in discovering new, naturally occurring, bioactive chemicals of plant origin for use in a variety of businesses, including the medical, pharmaceutical, and functional food sectors [3].

*Phoenix dactylifera* (date palm) is a flowering plant belonging to the palm family, Arecaceae, which is mostly cultivated for the consumption of its fruits. Although its original provenance is still disputed, its fruits are a staple cuisine for the Middle East and North Africa. Today, most of the world’s tropical and subtropical areas are normal habitats for date palm varieties. Additionally, date fruits are currently consumed as food in many regions of the world, particularly in Europe [4]. According to reports, date fruits have a total dietary fiber content ranging from 6.5 to 11.5% (up to 90% of which is insoluble and 10% of which is soluble), fat content of 1%, protein content of 2%, and ash content of 2%. Palm dates of diverse origins have been found to contain a variety of phenolic components with potent antioxidant activity [5]. Gallic acid, protocatechuic acid, p-coumaric acid, ferulic acid, *o*-coumaric acid, sinapic acid, certain cinnamic acid derivatives, flavonoids, and tannins are the primary components of dates [6].

In certain cultures, date fruit is used as traditional medicine to cure disorders such as digestive issues, fever, and bronchitis, as well as to heal wounds. Additional support for the therapeutic use of date fruits has been shown by recent studies. Preclinical research has proven that date fruits contain antibacterial and anti-inflammatory properties. However, further research is still required to assess the therapeutic validity of these findings. Similarly, studies about date fruits’ mechanisms of action are a necessity [7].

Nanotechnology has been applied in the medical sector since the beginning of the 21st century [8]. Several methods, including imaging, sensors, targeted medication and gene delivery systems, and artificial implants, are currently used to examine the most obscure areas of medical research. A mechanical procedure called ball milling makes it possible to carry out intentional physicochemical changes in powdered materials. The approach is supported by evidence that nonhydrostatic mechanical stresses and the resulting strains can impact the physical and chemical behaviors of individual molecules as well as those of ordered and disordered materials [9].

The current study was devoted to comparing and evaluating several highly edible cultivars of date palm (*Phoenix dactylifera*) in attempt to provide more in-depth information on their phenolic profiles and antioxidant and antimicrobial properties, as well as performing a combination study with some known antibiotics. Moreover, the most potent date palm extract was synthesized in a nano form through the ball-milling technique to enhance the observed biological activities.

## 2. Results and Discussion

### 2.1. Antimicrobial Effect of Date Extracts

Fresh and dried flesh of the fruit, fresh and dried epicarp, and seeds of Egyptian (“Zaghloul”, “Hayany”, and “Amhaat”) and Saudi (“Suqaey”, “Barhi”, and “Ajwa”) dates were extracted by methanol/chloroform (2:1) using the Soxhlet extraction technique. Each extract was tested against MDR microbes. Data shown in Table 1 and Table 2 revealed that the inhibition zone (IZ) diameters of Egyptian and Saudi date extracts ranged from 6.0 to 24.0 and from 6.0 to 10.5 mm, respectively, against the tested microbes. Therefore, Egyptian date extracts were chosen for further analyses. Moreover, the highest IZ diameter was found in fresh fruits of “Amhaat” date extract (FADE), followed by fresh fruits of “Hayany” dates (FHDE) and fresh fruits of “Zaghloul” dates (FZDE). The most resistant bacterium was *Escherichia coli*, followed by *Citrobacter freundii*, against all the tested Egyptian dates extracts. Moreover, data shown in Table 3 revealed that the minimum inhibitory concentration (MIC) and minimum bactericidal concentration (MBC) values of FADE, FZDE, and FHDE were in the ranges of 25–375 µg/mL, 25–1225 µg/mL, and 25–900 µg/mL, respectively, against all the tested MDR bacteria. Furthermore, the MIC indices of all the tested extracts against *Staphylococcus aureus*, *Escherichia coli*, and *Candida albicans* were less than 4, indicating that they exhibited bactericidal activities.

Many secondary metabolites extracted from plants have antibacterial activities [10,11]. The tested fruit and epicarp extracts demonstrated encouraging antibacterial activity against *S. pyogenes* [12]. Abuharfeil [12] demonstrated that date fruit extract inhibited streptolysin O’s hemolytic activity by neutralizing it, and 96% suppression was achieved at a relatively low dose. Date fruit, epicarp, and seed extract have been proven to be effective against Gram-negative bacteria (*Escherichia coli*) and Gram-positive bacteria (*Bacillus cereus*). The most resistant Gram-negative bacteria were *Shigella dysenteriae*, *Salmonella Typhi*, and *Klebsiella pneumoniae*, and the most resistant Gram-positive bacteria was *Staphylococcus aureus*, while the *Candida albicans* fungus was the most resistant against all the tested extracts of the palm date extracts [13].

### 2.2. Chemical Analysis of FADE, FHDE, and FZDE Extracts

#### 2.2.1. GC–MS Analysis

**Figure 1:**
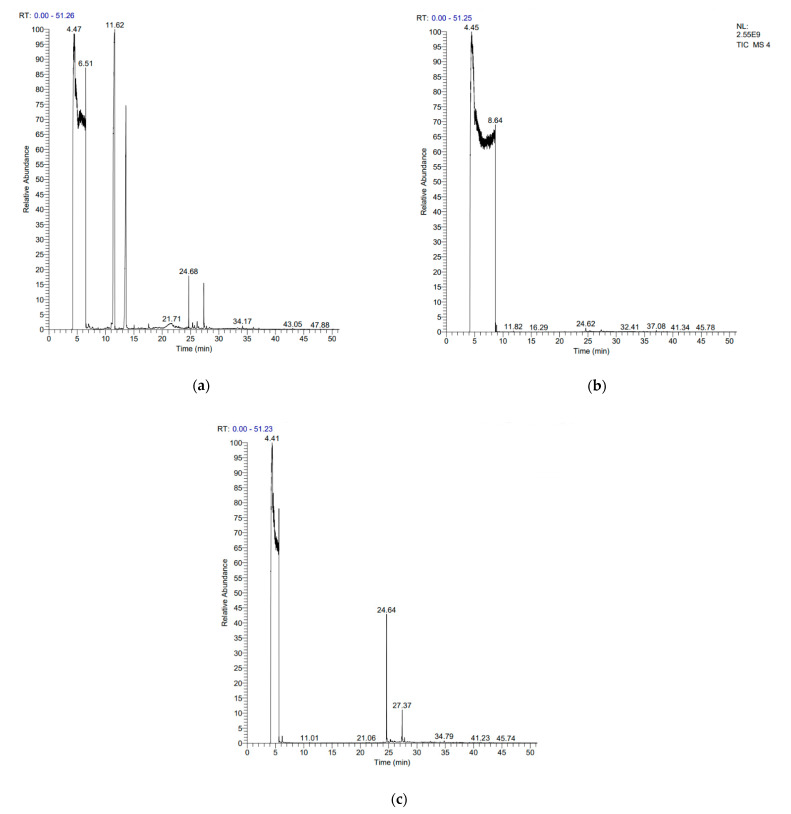
GC–MS chromatograms of FADE (**a**), FZDE (**b**), and FHDE (**c**) extracts.

FADE, FZDE and FHDE extracts were prepared and analyzed using GC–MS (Figure 1). In FADE extract, pentanoic acid, α-chloro-d-alanine, 2-furancarboxylic acid 3-methyl-methyl ester, and 1,2-benzenediol, *O*-(4-fluorobenzoyl)-*O*-(1-naphthoyl) were identified with 18.37, 28.70, 36.82, and 8.88 area percentages, respectively. In FZDE extract, only nicotinic acid was identified, with 72.44 area percentage. In FHDE extract, *N*-(phosphonomethyl) glycine, α-chloro-d-alanine, hexadecanoic acid methyl ester, and 9-octadecenoic acid (*Z*)-methyl ester were identified with 56.12, 31.60, 5.24, and 1.81 area percentages, respectively (Table 4).

Various levels of polyphenols and flavanols such as nicotinic acid, *p*-hydroxybenzoic acid, hexadecanoic acid, protocatechuic acid, pentanoic acid, vanillic acid, gallic acid, *O*-coumaric acid, and caffeic acid have been identified in palm date extracts [14]. Primary and secondary metabolite profiles of 21 date varieties from Egypt were analyzed by Farag et al. [15], who reported that caffeic acid conjugates (*O*-caffeoyl shikimic acid), flavonoids (quercetin and luteolin glycosides), sphingolipids, fatty acids, and flavonoids were detected as the most abundant classes amongst all the examined *P. dactylifera* fruit samples.

#### 2.2.2. Quantitative Determination of Total Phenolic, Flavonoid, and Tannin Contents

The total phenolic, flavonoid, and tannin contents in different extracts were determined using gallic acid, quercetin, and tannic acid as standards, respectively. The absorbance values obtained at different concentrations were used for the construction of calibration curves (Table S1, Supplementary Materials). The total phenolic, flavonoid, and tannin contents of the extracts were calculated from the regression equations of the calibration curves:

Y = 0.0019x−0.0068, R² = 0.9935; Y = 0.0027x + 0.0401, R² = 0.9005; and Y = 0.0036x + 0.0468, R² = 0.9361, respectively (Figure S1). The maximum total phenolic, flavonoid, and tannin contents were found in FHDE extract at 538.578 µg/mL, 28.481 µg/mL, and 20.888 µg/mL, respectively, followed by FADE (506.210 µg/mL, 16.259 µg/mL, and 14.777 µg/mL, respectively) and FZDE (174.105 µg/mL, 3.851 µg/mL, and 2.277 µg/mL, respectively) (Table 5).

The observed differences in phenolic component composition could have resulted from the differences in date cultivars, solvents, extraction techniques, and geographic location, which may have caused the observed antibacterial activity variance across the tested date extracts [14]. Authors who have studied the antibacterial properties of date extracts have also noted the presence of polyphenols, flavonoids, and tannins, which together have an antioxidant effect. Another important pharmacological activity is the inhibition of microbial growth by these secondary metabolites, which can be useful in powerful natural products and phytochemicals [16]. These compounds may act individually as a biologically active compounds or provide a synergistic effect when added to antibiotics to achieve higher antibacterial effect [17]. Several date cultivars were extracted and assessed for their total flavonoid and tannin concentrations, which were in the ranges of 0.0135–2.99 µg/mL and 14.654–27.422 µg/mL, respectively [18].

Ibourki et al. [19] assessed eight different types of Moroccan date palms. “Boufgous”, “Agondari”, and “Bouskri” had the highest weight percentages for whole fruit, flesh, and seeds, respectively. Proteins (1.60–3.53%), moisture (5.31–17.31%), ash (2.08–2.50%), lipids (0.32–1.09%), carbohydrates (76.69–90.18%), energy value (338.30–385.89 kcal/100 g), and reducing capacity (100.14–1607.12 mg GAE/100 g) were the fruit flesh’s greatest advantages.

#### 2.2.3. Antioxidant Activity

**Figure 2:**
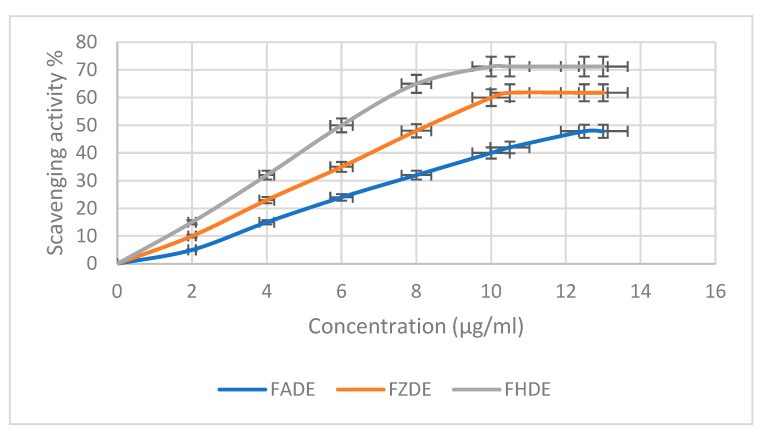
Antioxidant activity of FADE, FHDE, and FZDE extracts.

The scavenging activity of FADE, FHDE, and FZDE extracts against 2,2-diphenyl-1-picrylhydrazyl (DPPH) radicals increased in a concentration-dependent way (Figure 2). The maximum scavenging activity was shown by FHDE extract at 71.21%, with IC50 10.16 µg/mL, followed by FZDE (61.75%; IC50 10.45 µg/mL) then FADE (47.85%; IC50 12.49 µg/mL) extracts (Table 6). Similarly, Mrabet et al. [20] showed that date extracts had strong radical scavenging activity, with IC50 values in the range of 1.65–8.25 μg/mL, by using the DPPH antioxidant method [20]. Another study revealed that the tested date extracts showed good DPPH radical scavenging (IC50 103–177 μg/mL) and hydroxyl radical scavenging (IC50 1.1–1.55 mg/mL) activity and total antioxidant capacity (IC50 87–192 μg/mL). The significant variation in the degree of antioxidant activity can be attributed to the differences in the content and composition of phenolic compounds [21]. Similar results were observed with spent coffee grounds (SCGs), which showed high antioxidant activity as assessed by DPPH (up to 92.12 ± 0.22%) and DPPH IC50 (up to 53.73 ± 1.03 µg mL^−1^) [22].

**Figure 3:**
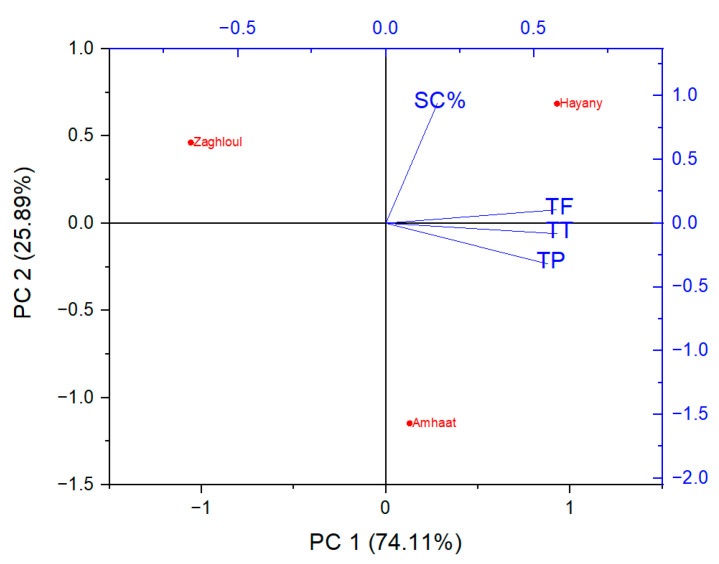
Principal component analysis (PCA) projections on PC1 and PC2. Plotted points are mean values of each studied parameter of extracts from the fresh fruits of “Zaghloul”, “Hayany”, and “Amhaat” Egyptian dates. TF: total flavonoids, TP: total phenolics, TT: total tannins, SC%: DPPH scavenging activity (%).

Hence, principal component analysis (PCA) was used as a multivariate statistical method to be an efficient discriminative approach to differentiate among the date palm varieties and the observed scavenging and phytochemical analyses (dependent variables). The first two principal components (PCs) explained over 74% of the total data variabilities. The first component (PC1) seemed to separate between the tested dates extracts and the observed phytochemical analyses (Figure 3). Fresh fruit of “Amhaat” and “Hayany” date extracts were on the positive side of the first component (PC1), while “Zaghloul” date extract was on the opposite side. Furthermore, total flavonoids (TF), total phenolics (TP), and total tannins (TT) were linked and related as dependent variables.

Ibourki et al. [19] mentioned that “Ismmarti”, “Boufgous”, “Amouch”, and “Jihel” interacted, with higher scores of seed weight, fruit weight, flesh weight, and flesh percentage, while “Ismmarti”, “Agondari”, and “Boufgous” interacted with greater values of reducing capacity and mineral elements.

### 2.3. Effects of Combinations of FADE, FHDE, and FZDE Extracts with Commonly Used Antibiotics

The interactions of FADE, FHDE, and FZDE with commonly known antibiotics were evaluated against *C. albicans*, *E. coli*, and *S. aureus* individually using disc diffusion method. Data shown in Table 7 revealed that the combined actions of FHDE with amikacin (AK), ampicillin/cloxacillin (AX), amoxicillin/clavulanate (AMC), gentamicin (GEN), and cloxacillin (COX) showed synergistic effects against *C. albicans*, while the combinations with AK and COX showed synergistic action against *S. aureus*. In addition, when polymyxin B (PB) was combined with FHDE, it showed an indifferent effect against *S. aureus*. Furthermore, FZDE showed a synergistic effect against *E. coli* when combined with COX. However, other combinations showed antagonistic effects. The most potent combination against the tested pathogens was FHDE combined with AK. Therefore, this combination was subjected to further investigations.

An efficient strategy for dealing with the resistance problem is the synergistic interaction of natural substances with commonly known antibiotics. The interaction of an antimicrobial agent with its target within the pathogen may be improved or designed to facilitate a combination between natural substances, which would stop the development of resistance. Because it allows for the use of lower doses of both drugs, this technique can minimize drug toxicity [23,24]. The emergence of microbial resistance to antibiotics has made it incredibly challenging to identify new treatments. Abreu et al. [24] and Betts et al. [25] isolated some phytochemicals from herbs that were biologically active. For instance, reserpine, isopimarane, EGCG, and carnosic acid blocked bacterial efflux pumps, whereas corilagin, gallic acid, and thymol increased the permeability of bacterial outer membranes. Tellimagrandin and corilagin both inhibited the penicillin binding proteins (PBP2a) [24]. Berberine and 5′-methoxyhydnocarpin, two phytochemicals typically found in barberry plants, worked together to concentrate inside bacteria and inhibit the MDR efflux pump [24]. Chaqroune and Taleb [26] declared that Soxhlet extraction combined with polar solvents (ethanol and methanol) led to the best MIC (8.17 ± 1.04–24.20 ± 0.98 μg/mL) and IC50 values (50.02 ± 0.08–390.00 ± 1.00 µg/mL) obtained from wild rosemary.

### 2.4. Nano-Date Palm Fruit Extract Synthesis and Characterization

**Figure 4:**
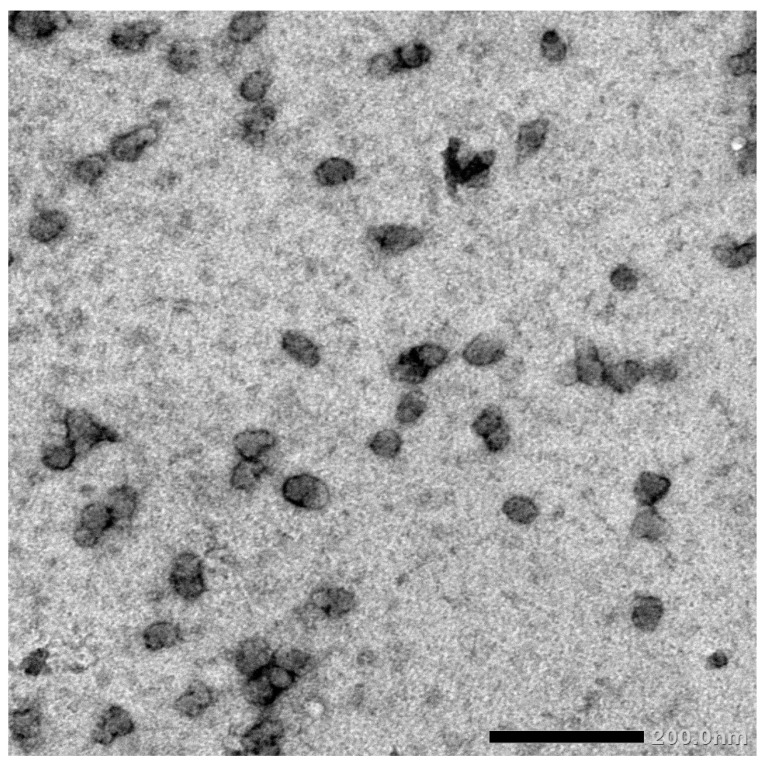
TEM micrograph of the synthesized NFHDE/AK.

FHDE nanoparticles were physically synthesized using the ball-milling technique. Then, the synthesized nano-FHDE (NFHDE) was combined with AK (FHDE/AK, 1:1 *w*/*w*). TEM images of the nano-FHDE/AK (NFHDE/AK) showed it to comprise homogenous unilamellar vesicles. These were well identified as having nearly perfect sphere-like shapes and smooth vesicle surfaces (Figure 4). The recorded vesicle size was 21.6 nm, while the PDI and zeta potential were 0.32 and +38.4 mV, respectively, which proved the nanoparticles’ homogeneity and stability. Alothman et al. [27] revealed that nano-date extract was a heterogeneous mix of spherical and irregular shapes. Evidently, the particles’ sphere-like structure may provide them highly maintained flexibility for useful applications. According to TEM investigations, the size range was between 30 and 110 nm. Similarly, Saba et al. [28] reported that the nanosize of date fruit (using the ball-milling process) was up to 100 nm.

### 2.5. Antimicrobial Activity of the Synthesized NFHDE/AK Extract

**Figure 5:**
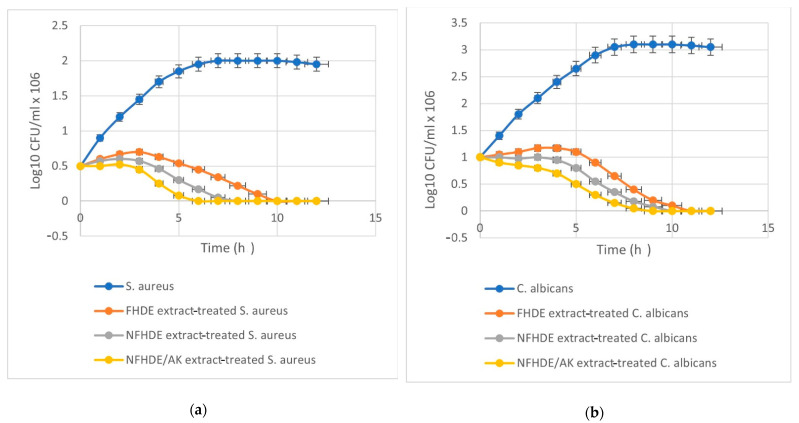
Time kill curve of FHDE and NFHDE/AK extract treated *S. aureus* (**a**) and *C. albicans* (**b**) cells.

**Figure 6:**
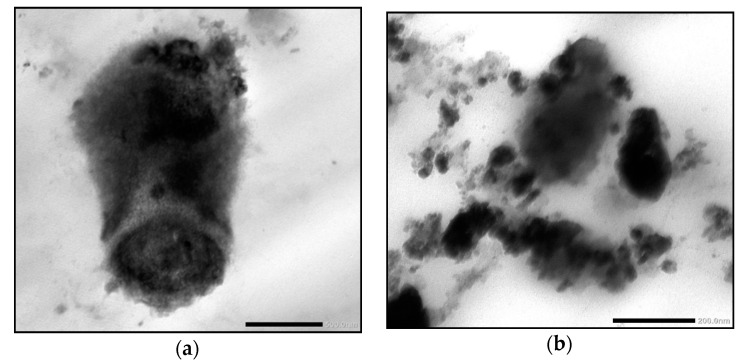
Transmission electron micrographs of NFHDE/AK-treated *S. aureus* (**a**) and *C. albicans* (**b**) cells.

Data shown in Table 8 revealed that the inhibition zone (IZ) diameter of NFHDE/AK against *C. albicans* was 38 mm, with an MIC, MBC, and MIC index of 25 µg/mL, 50 µg/mL and 2, respectively. Its IZ diameter against *S. aureus* was 34 mm, with an MIC, MBC, and MIC index of 12.5 µg/mL, 25 µg/mL, and 2, respectively. In order to test the eradication time for FHDE- and NFHDE/AK-treated *C. albicans* and *S. aureus* cells, microbial lethality curves were evaluated. The results, as shown in Figure 5, showed a steady increase in the growth of *S. aureus* and *C. albicans* during the log phase (first 7 h and 8 h, respectively) after which microbial growth sharply decreased until complete eradication after 6 h and 9 h for NFHDE/AK formula-treated *S. aureus* and *C. albicans* cells respectively. When the microbial cells were treated with FHDE, the cells were eradicated after 10 h. Transmission electron microscopy (TEM) was applied to NFHDE/AK-treated *S. aureus* and *C. albicans* cells. Figure 6a,b shows that the microbial cells treated with NFHDE/AK revealed excessive damage to the cell wall and huge leakage of intracellular components that led to cell death. The nanoparticles of date extract were able to reduce Gram-positive and Gram-negative bacterial growth by disrupting the cell membrane with 100-fold higher antibacterial activity than the crude palm date extract [29].

## 3. Materials and Methods

### 3.1. Sample Collection

Date palm fruits were collected at the “Tamr stage” (full ripeness) from local markets in Saudi Arabia and Egypt during September 2021. The local Arabic names of the cultivars used in this study were “Suqaey”, “Barhi”, and “Ajwa” (red, yellow, and brown, respectively) for the Saudi Arabian dates and “Zaghloul”, “Hayany”, and “Amhaat” (red, yellow, and brown, respectively) for the Egyptian dates. Samples were cleaned and rinsed with sterile distilled water, air dried, and ground into powder before being conserved at −20 °C.

### 3.2. Extraction of Bioactive Material

The conventional Soxhlet method was used for the extraction of bioactive agents from the fresh fruit flesh, dried fruit flesh, epicarp, dried epicarp, and seeds of different Egyptian and Saudi date palm cultivars. One at a time, 15 g of each of the powdered dates was extracted by methanol/chloroform (2:1) [30].

### 3.3. Microorganisms

*Pseudomonas aeruginosa*, *Acinetobacter baumannii*, *Proteus vulgaris*, *Staphylococcus aureus*, *Citrobacter freundii*, *Escherichia coli*, *Enterobacter aerogenes*, *Candida albicans*, and *Klebsiella pneumonia* (Table 9) were kindly provided and identified from El-Shatby Pediatric Hospital (Alexandria, Egypt). Each strain was kept in brain–heart infusion glycerol broth at −4 °C until testing.

### 3.4. Antibacterial Effect of Date Extracts

Antimicrobial activity was investigated using the disk diffusion method according to CLSI guidelines [31]. By measuring the minimum inhibitory concentration (MIC), minimum bactericidal concentration (MBC), and microbial lethality curve, further antibacterial activity was evaluated [32].

### 3.5. Chemical Analysis of the Most Promising Extracts

#### 3.5.1. GC–MS (Gas Chromatography–Mass Spectroscopy) Analysis

The chemical analysis and component identification of the date extract using GC–MS analysis were performed according to Hamza et al. [33].

#### 3.5.2. Phytochemical Estimation of Antioxidant Components (Total Phenolic, Flavonoid, and Tannin Content)

Using gallic acid, quercetin, and tannic acid as standards, the total phenolic content (TPC), total flavonoid content (TFC), and total tannin content (TTC), respectively, of the date extracts were measured [21,34]. According to standard curve calculations, the findings of the TPC, TFC, and TTC contents are represented as gallic acid equivalent (mg GAE/g), quercetin equivalent (mg QE/g), and tannic acid equivalent (mg TAE/g), respectively.

#### 3.5.3. DPPH Free Radical Scavenging Assay

The technique of Zihad et al. [21] was used to assess the 2,2-diphenyl-1-picrylhydrazyl (DPPH) free radical scavenging activity of the date extracts. Aliquots (50 µL) of serially diluted extract solutions in methanol (500–0.98 µg/mL) were combined with 5 mL of DPPH solution in methanol (40 µg/mL). The reaction mixture was completely vortexed and kept at room temperature for 30 min in the dark. After then, a UV spectrophotometer (Shimadzu 2000) was used to assess the mixture’s absorbance at 517 nm. Percent inhibition was obtained using ascorbic acid as the positive control using Equation (1):

(1) $$\%\;\mathrm{Inhibition}=\lfloor 1-\frac{\mathrm{Abs}\;\mathrm{Sample}}{\mathrm{Abs}\;\mathrm{Control}}\rfloor \times 100$$

where Abs control is the absorbance of the DPPH solution dissolved in methanol and Abs sample is the absorbance of the tested date extract or the standard solution dissolved in methanol.

#### 3.5.4. Total Antioxidant Capacity

For this experiment, a modified version of Yang et al.’s technique was applied [35]. In this test, 3 mL of the reagent mixture (4 mM ammonium molybdate, 0.6 M sulfuric acid, and 28 mM trisodium phosphate) was blended with 0.3 mL of the tested date extract or a standard solution (ascorbic acid). This reaction mixture was heated to 90 °C and then allowed to cool to room temperature for 90 min. At 695 nm, the reaction mixture’s absorbance was appraised by comparing the absorbance of the blank preparation with the IC50 values.

##### Statistical Analyses

Results are expressed as means ± standard deviations (SD). Principal component analysis (PCA) was performed to differentiate among date extract varieties and the estimated phytochemicals using ORIGINPro^®^ 2022b (OriginLab, Inc., Northampton, MA, USA).

### 3.6. Effects of Combinations of the of the Most Promising Date Extracts with Commonly Used Antibiotics Assessed Using the Disk Diffusion Method

Different antibiotics were selected in the present experiment, namely, ceftriaxone (CTR, 30 μg), amikacin (AK, 30 μg), amoxicillin/clavulanate (AMC, 20/10 µg), gentamicin (GEN, 10 µg), ampicillin/cloxacillin (AX, 10 µg), cloxacillin (COX, 5 µg), ciprofloxacin (CIP, 5 µg), oxacillin (OX, 1 µg), polymyxin (PB, 5 µg), and cotrimoxazole (COT, 25 µg). The antibiotic discs were loaded with 20 µL of each date extract (20 µg) one at a time and placed on the surface of the inoculated Mueller–Hinton agar. When the combined effect was equal to the sum of the individual effects, the action was considered as additive. Antagonism was observed when the effect of the combined compounds was less than that of the individual compounds. Synergism was observed when the effect of the combined compounds was greater than the sum of the individual effects, while indifference was observed in the absence of interaction [36].

### 3.7. Physical Synthesis of Nanoparticles of the Most Promising Extract Using Ball-Milling Technique

With slight modifications, the milling procedure was carried out in accordance with the methodology of Saba et al. [28]. Using 10 mm zirconia balls in a 15 mL zirconia container, the date extract-to-ball ratio was maintained at 1:10. An entire number of 30 min cycles was maintained, with a 15 min break in between each cycle. The RPM was maintained at 300 throughout the operation.

### 3.8. Characterization of the Synthesized Nanoextract

The prepared nanoparticles were mixed in a constant ratio that equaled 1:1 (*w*/*w*) with the most potent antibiotic (which proved promising synergistic activity with the crude date extract). The potent formula was characterized through dynamic light scattering (DLS) techniques to determine the vesicle size, polydispersity index (PDI), and zeta potential. The ultrastructure of the prepared nanoparticles was analyzed through transmission electron microscopic (TEM) study. Moreover, the antibacterial activity of the prepared nanoparticles was evaluated using the disk diffusion method via the minimal inhibitory concentration (MIC), minimum bactericidal concentration (MBC), and microbial lethality curve along with TEM examination of the treated microbial cells [37].

## 4. Conclusions

According to the data provided, it was concluded that Egyptian date extracts (“Zaghloul”, “Hayany”, and “Amhaat” (red, yellow, and brown Egyptian dates)) showed high antimicrobial activity against all of the tested multidrug resistant microbes, especially *Candida albicans* and *Staphylococcus aureus*. The extract of the fresh fruit of “Hayany” (yellow) dates showed the highest antimicrobial activities, with a promising antioxidant activity and the highest concentrations of total phenolics, flavonoids, and tannins. The combination between the extract of the fresh fruit of “Hayany” dates and amikacin had synergistic activity. The novel nanoformula (NFHDE/AK, nano-fresh fruit of “Hayany” date extract/amikacin) showed the highest antimicrobial activity. Egyptian date extracts can be used as bioactive healthy compound sources. However, extensive preclinical and clinical studies must be undertaken for the benefit of humankind.

## Figures and Tables

**Table 1:** Antimicrobial effects of Egyptian date extracts against some pathogens.

Test Organism	Inhibition Zone Diameter (mm) ± SD														
	“Zaghloul” Date					“Hayany” Date					“Amhaat” Date				
	Fruit	Dried Fruit	Epicarp	Dried Epicarp	Seed	Fruit	Dried Fruit	Epicarp	Dried Epicarp	Seed	Fruit	Dried Fruit	Epicarp	Dried Epicarp	Seed
*Pseudomonas aeruginosa*	10.5 ± 0.1	6.0 ± 0.3	6.0 ± 0.2	6.0 ± 0.5	6.0 ± 0.2	10.0 ± 0.4	9.0 ± 0.6	6.0 ± 0.3	6.0 ± 0.1	6.0 ± 0.5	15.5 ± 0.1	6.0 ± 0.2	6.0 ± 0.4	6.0 ± 0.3	6.0 ± 0.2
*Acinetobacter baumannii*	9.0 ± 0.4	6.0 ± 0.2	6.0 ± 0.1	6.0 ± 0.3	6.0 ± 0.1	13.0 ± 0.8	6.0 ± 0.3	6.0 ± 0.6	6.0 ± 0.1	6.0 ± 0.3	20.0 ± 0.1	6.0 ± 0.1	6.0 ± 0.1	6.0 ± 0.6	6.0 ± 0.1
*Proteus vulgaris*	11.0 ± 0.1	9.5 ± 0.2	8.0 ± 0.1	8.0 ± 0.2	7.5 ± 0.4	11.5 ± 0.6	9.5 ± 0.6	6.5 ± 0.8	6.5 ± 0.2	6.5 ± 0.5	19.0 ± 0.7	0.6 ± 0.4	6.0 ± 0.3	6.0 ± 0.8	6.0 ± 0.4
*Staphylococcus aureus*	11.0 ± 0.1	9.5 ± 0.3	6.0 ± 0.3	6.0 ± 0.1	6.0 ± 0.6	13.5 ± 0.3	6.0 ± 0.2	6.0 ± 0.2	6.0 ± 0.2	6.0 ± 0.8	20.0 ± 0.2	6.0 ± 0.6	6.0 ± 0.3	6.0 ± 0.2	6.0 ± 0.6
*Citrobacter freundii*	9.0 ± 0.4	6.0 ± 0.3	6.0 ± 0.3	6.0 ± 0.9	6.0 ± 0.8	8.0 ± 0.2	7.0 ± 0.7	6.0 ± 0.1	6.0 ± 0.9	6.0 ± 0.2	24.0 ± 0.9	6.0 ± 0.8	6.0 ± 0.5	6.0 ± 0.1	6.0 ± 0.8
*Escherichia coli*	8.5 ± 0.8	6.0 ± 0.1	6.0 ± 0.1	6.0 ± 0.6	6.0 ± 0.2	8.0 ± 0.9	6.0 ± 0.1	6.0 ± 0.2	6.0 ± 0.1	6.0 ± 0.2	9.5 ± 0.3	6.0 ± 0.2	6.0 ± 0.8	6.0 ± 0.2	6.0 ± 0.2
*Enterobacter aerogenes*	14.0 ± 0.2	13.5 ± 0.8	11.0 ± 0.7	11.0 ± 0.8	6.0 ± 0.1	15.0 ± 0.2	11.0 ± 0.6	6.0 ± 0.1	6.0 ± 0.4	6.0 ± 0.1	22.0 ± 0.5	6.0 ± 0.1	6.0 ± 0.9	6.0 ± 0.1	6.0 ± 0.1
*Candida albicans*	13.0 ± 0.9	6.0 ± 0.4	6.0 ± 0.4	6.0 ± 0.3	6.0 ± 0.5	11.0 ± 0.2	9.0 ± 0.1	6.0 ± 0.5	6.5 ± 0.3	6.5 ± 0.1	18.0 ± 0.3	6.0 ± 0.5	6.0 ± 0.1	6.0 ± 0.5	6.0 ± 0.5
*Klebsiella pneumoniae*	13.0 ± 0.1	8.5 ± 0.1	6.0 ± 0.5	6.0 ± 0.7	6.0 ± 0.1	7.5 ± 0.6	6.0 ± 0.2	6.0 ± 0.3	6.0 ± 0.1	6.0 ± 0.9	17.5 ± 0.7	6.0 ± 0.1	6.0 ± 0.8	6.0 ± 0.3	6.0 ± 0.1

SD: standard deviation.

**Table 2:** Antimicrobial effects of Saudi Arabian date extracts against some pathogens.

Test Organism	Inhibition Zone Diameter (mm) ± SD														
	“Suqaey” Date					“Barhi” Date					“Ajwa” Date				
	Fruit	Dried Fruit	Epicarp	Dried Epicarp	Seed	Fruit	Dried Fruit	Epicarp	Dried Epicarp	Seed	Fruit	Dried Fruit	Epicarp	Dried Epicarp	Seed
*Pseudomonas aeruginosa*	12.5 ± 0.2	6.0 ± 0.3	6.0 ± 0.2	6.0 ± 0.4	6.0 ± 0.8	6.0 ± 0.1	6.0 ± 0.3	11.0 ± 0.2	6.0 ± 0.4	6.0 ± 0.8	10.0 ± 0.1	6.0 ± 0.3	6.0 ± 0.4	6.0 ± 0.8	6.0 ± 0.3
*Acinetobacter baumannii*	10.0 ± 0.3	6.0 ± 0.2	9.0 ± 0.1	6.0 ± 0.3	6.0 ± 0.1	6.0 ± 0.2	6.0 ± 0.2	6.0 ± 0.4	6.0 ± 0.3	6.0 ± 0.1	10.5 ± 0.5	6.0 ± 0.2	6.0 ± 0.3	6.0 ± 0.1	6.0 ± 0.2
*Proteus vulgaris*	9.0 ± 0.6	6.0 ± 0.4	7.0 ± 0.2	6.0 ± 0.2	8.5 ± 0.3	6.0 ± 0.4	6.0 ± 0.4	10.0 ± 0.7	6.0 ± 0.2	6.0 ± 0.3	7.0 ± 0.2	6.0 ± 0.4	6.0 ± 0.2	6.0 ± 0.3	6.0 ± 0.4
*Staphylococcus aureus*	6.0 ± 0.2	6.0 ± 0.3	7.5 ± 0.2	6.0 ± 0.1	6.0 ± 0.4	6.0 ± 0.1	6.0 ± 0.3	6.0 ± 0.2	6.0 ± 0.1	6.0 ± 0.4	10.5 ± 0.2	6.0 ± 0.3	6.0 ± 0.1	6.0 ± 0.4	6.0 ± 0.3
*Citrobacter freundii*	9.0 ± 0.7	6.0 ± 0.2	6.0 ± 0.4	6.0 ± 0.2	6.0 ± 0.1	6.0 ± 0.9	6.0 ± 0.2	6.0 ± 0.1	6.0 ± 0.2	6.0 ± 0.1	9.0 ± 0.1	6.0 ± 0.2	6.0 ± 0.2	6.0 ± 0.1	6.0 ± 0.2
*Escherichia coli*	9.0 ± 0.2	6.0 ± 0.2	9.0 ± 0.7	6.0 ± 0.1	6.0 ± 0.1	6.0 ± 0.6	6.0 ± 0.2	6.0 ± 0.8	6.0 ± 0.1	6.0 ± 0.1	10.5 ± 0.9	6.0 ± 0.2	6.0 ± 0.1	6.0 ± 0.1	6.0 ± 0.2
*Enterobacter aerogenes*	6.0 ± 0.5	6.0 ± 0.4	6.0 ± 0.2	6.0 ± 0.3	6.0 ± 0.2	6.0 ± 0.2	6.0 ± 0.4	6.0 ± 0.3	6.0 ± 0.3	6.0 ± 0.2	9.0 ± 0.3	6.0 ± 0.4	6.0 ± 0.3	6.0 ± 0.2	6.0 ± 0.4
*Candida albicans*	8.0 ± 0.4	6.0 ± 0.3	6.0 ± 0.9	6.0 ± 0.1	6.0 ± 0.4	8.0 ± 0.3	6.0 ± 0.3	6.0 ± 0.2	6.0 ± 0.1	6.0 ± 0.4	7.5 ± 0.1	6.0 ± 0.3	6.0 ± 0.1	6.0 ± 0.4	6.0 ± 0.3
*Klebsiella pneumoniae*	9.0 ± 0.3	6.0 ± 0.2	7.0 ± 0.1	6.0 ± 0.1	6.0 ± 0.2	6.0 ± 0.6	6.0 ± 0.2	6.0 ± 0.9	6.0 ± 0.1	6.0 ± 0.2	10.0 ± 0.2	6.0 ± 0.2	6.0 ± 0.1	6.0 ± 0.2	6.0 ± 0.2

SD: standard deviation.

**Table 3:** MIC, MBC, and MIC indices of the most promising Egyptian date extracts.

Tested Extract	FZDE			FHDE			FADE		
	MIC (µg/mL)	MBC (µg/mL)	MIC Index	MIC (µg/mL)	MBC (µg/mL)	MIC Index	MIC (µg/mL)	MBC (µg/mL)	MIC Index
*Pseudomonas aeruginosa*	150.0	750.0	5.0	125.0	750.0	6.0	75.0	375.0	5.0
*Acinetobacter baumannii*	175.0	1225.0	7.0	100.0	500.0	5.0	50.0	300.0	6.0
*Proteus vulgaris*	200.0	1200.0	6.0	150.0	900.0	6.0	75.0	375.0	5.0
*Staphylococcus aureus*	25.0	50.0	2.0	25.0	25.0	1.0	50.0	50.0	1.0
*Citrobacter freundii*	200.0	1000.0	5.0	175.0	875.0	5.0	50.0	250.0	5.0
*Escherichia coli*	50.0	150.0	3.0	75.0	150.0	2.0	75.0	150.0	2.0
*Enterobacter aerogenes*	150.0	750.0	5.0	100.0	600.0	6.0	50.0	250.0	5.0
*Candida albicans*	75.0	150.0	2.0	75.0	75.0	1.0	25.0	50.0	2.0
*Klebsiella pneumoniae*	100.0	600.0	6.0	150.0	900.0	6.0	50.0	250.0	5.0

MIC: minimum inhibitory concentration, MBC: minimal bactericidal concentration, FZDE: fresh fruit of “Zaghloul” date extract, FHDE: fresh fruit of “Hayany” date extract, FADE: fresh fruit of “Amhaat” date extract.

**Table 4:** The major expected compounds of Egyptian fresh date extracts and their structures.

Extract	Proper Compound	RT	Probability	Area%	Structure
FADE	Pentanoic acid	6.50	63.71	18.37	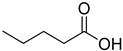
	α-Chloro-d-alanine	4.31	89.23	28.70	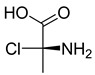
	2-Furancarboxylic acid, 3-methyl-, methyl ester	11.61	15.59	36.82	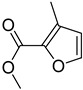
	1,2-Benzenediol, *O*-(4-fluorobenzoyl)-*O*-(1-naphthoyl)	13.59	13.22	8.88	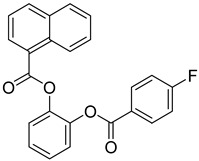
FZDE	Nicotinic acid	4.37	92.12	72.44	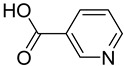
FHDE	*N*-(phosphonomethyl) glycine	4.24	96.90	56.12	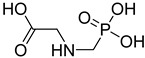
	α-Chloro-d-alanine	5.58	46.58	31.60	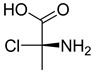
	Hexadecanoic acid, methyl ester	24.64	56.02	5.24	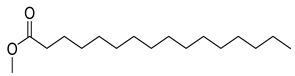
	9-Octadecenoic acid (*Z*), methyl ester	27.37	37.08	1.81	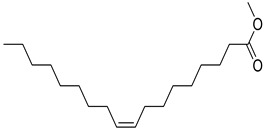

FZDE: fresh fruit of “Zaghloul” date extract, FHDE: fresh fruit of “Hayany” date extract, FADE: fresh fruit of “Amhaat” date extract.

**Table 5:** Total phenolic, flavonoid, and tannin concentrations of the tested extracts.

Samples		FADE	FZDE	FHDE
Total phenolics	Absorbance	1.935 ± 0.52	0.974 ± 0.09	1.151 ± 0.21
	Blank	0.955 ± 0.11	0.324 ± 0.07	1.016 ± 0.32
	Concentration (µg/mL)	506.210 ± 11.03	174.105 ± 7.01	538.578 ± 17.06
Total flavonoids	Absorbance	0.280 ± 0.08	0.125 ± 0.07	0.215 ± 0.09
	Blank	0.084 ± 0.01	0.050 ± 0.01	0.117 ± 0.06
	Concentration (µg/mL)	16.259 ± 1.07	3.851 ± 0.91	28.481 ± 3.04
Total tannins	Absorbance	0.231 ± 0.13	0.255 ± 0.09	1.002 ± 0.95
	Blank	0.100 ± 0.07	0.055 ± 0.01	0.122 ± 0.09
	Concentration (µg/mL)	14.777 ± 2.93	2.277 ± 0.98	20.888 ± 4.72

FZDE: fresh fruit of “Zaghloul” date extract, FHDE: fresh fruit of “Hayany” date extract, FADE: fresh fruit of “Amhaat” date extract.

**Table 6:** Antioxidant activity of FADE, FHDE, and FZDE extracts.

Sample	IC50 (µg/mL)	DPPH Scavenging Activity (%)
FADE	12.49	47.85
FZDE	10.45	61.75
FHDE	10.16	71.21

FZDE: fresh fruit of “Zaghloul” date extract, FHDE: fresh fruit of “Hayany” date extract, FADE: fresh fruit of “Amhaat” date extract.

**Table 7:** Effects of combinations of date extract with commonly used antibiotics.

		Inhibition Zone Diameter (mm) ± SD		
Antibiotic	Combination With	*C. albicans*	*E. coli*	*S. aureus*
Amikacin AK	Alone	6.0 ± 0.2	19.0 ± 0.1	6.0 ± 0.6
	+FADE	6.0 ± 0.1	23.0 ± 0.2	23.0 ± 0.2
	+FZDE	6.0 ± 0.3	24.0 ± 0.2	15.0 ± 0.1
	+FHDE	27.0 ± 0.8	30.0 ± 0.5	22.0 ± 0.3
Ampicillin/cloxacillin AX	Alone	6.0 ± 0.2	6.0 ± 0.6	6.0 ± 0.5
	+FADE	6.0 ± 0.2	14.0 ± 0.2	6.0 ± 0.2
	+FZDE	6.0 ± 0.1	6.0 ± 0.9	6.0 ± 0.5
	+FHDE	28.0 ± 0.1	6.0 ± 0.7	6.0 ± 0.7
Amoxicillin/clavulanate AMC	Alone	6.0 ± 0.2	6.0 ± 0.5	23.0 ± 0.9
	+FADE	6.0 ± 0.7	6.0 ± 0.5	22.0 ± 0.2
	+FZDE	6.0 ± 0.8	14.0 ± 0.7	6.0 ± 0.2
	+FHDE	20.0 ± 0.3	10.0 ± 0.2	6.0 ± 0.3
Gentamicin GEN	Alone	6.0 ± 0.7	16.0 ± 0.2	6.0 ± 0.2
	+FADE	6.0 ± 0.2	19.0 ± 0.1	6.0 ± 0.5
	+FZDE	6.0 ± 0.3	20.0 ± 0.1	6.0 ± 0.2
	+FHDE	20.0 ± 0.1	16.0 ± 0.1	19.0 ± 0.5
Cloxacillin COX	Alone	6.0 ± 0.2	6.0 ± 0.5	12.0 ± 0.6
	+FADE	6.0 ± 0.1	6.0 ± 0.8	6.0 ± 0.9
	+FZDE	6.0 ± 0.3	15.0 ± 0.5	6.0 ± 0.8
	+FHDE	22.0 ± 0.3	10.0 ± 0.3	28.0 ± 0.5
Ciprofloxacin CIP	Alone	36.0 ± 0.9	38.0 ± 0.4	11.0 ± 0.2
	+FADE	6.0 ± 0.5	40.0 ± 0.8	16.0 ± 0.1
	+FZDE	6.0 ± 0.8	28.0 ± 0.3	14.0 ± 0.1
	+FHDE	36.0 ± 0.3	22.0 ± 0.8	17.0 ± 0.4
Oxacillin OX	Alone	12.0 ± 0.2	6.0 ± 0.1	6.0 ± 0.5
	+FADE	6.0 ± 0.3	11.0 ± 0.2	6.0 ± 0.2
	+FZDE	6.0 ± 0.1	11.0 ± 0.2	6.0 ± 0.1
	+FHDE	17.0 ± 0.1	6.0 ± 0.1	6.0 ± 0.3
Polymyxin PB	Alone	14.0 ± 0.9	16.0 ± 0.2	6.0 ± 0.2
	+FADE	17.0 ± 0.5	19.0 ± 0.3	6.0 ± 0.2
	+FZDE	20.0 ± 0.4	15.0 ± 0.4	6.0 ± 0.9
	+FHDE	18.0 ± 0.6	15.0 ± 0.4	17.0 ± 0.8
Ceftriaxone CTR	Alone	13.0 ± 0.2	35.0 ± 0.1	20.0 ± 0.2
	+FADE	6.0 ± 0.2	42.0 ± 0.5	21.0 ± 0.5
	+FZDE	6.0 ± 0.1	30.0 ± 0.2	6.0 ± 0.3
	+FHDE	15.0 ± 0.2	32.0 ± 0.4	6.0 ± 0.3
Cotrimoxazole COT	Alone	39.0 ± 0.9	23.0 ± 0.2	6.0 ± 0.2
	+FADE	6.0 ± 0.5	32.0 ± 0.6	6.0 ± 0.2
	+FZDE	6.0 ± 0.1	20.0 ± 0.8	6.0 ± 0.1
	+FHDE	47.0 ± 0.2	25.0 ± 0.5	6.0 ± 0.2

FZDE: fresh fruit of “Zaghloul” date extract, FHDE: fresh fruit of “Hayany” date extract, FADE: fresh fruit of “Amhaat” date extract.

**Table 8:** Antimicrobial activity of the synthesized NFHDE/AK extract.

Pathogen	IZ (mm)	MIC (µg/mL)	MBC (µg/mL)	MIC Index
*Candida albicans*	38	25	50	2
*Staphylococcus aureus*	34	12.5	25	2

IZ: inhibition zone diameter, MIC: minimum inhibitory concentration, MBC: minimal bactericidal concentration, FZDE: fresh fruit of “Zaghloul” date extract, FHDE: fresh fruit of “Hayany” date extract, FADE: fresh fruit of “Amhaat” date extract.

**Table 9:** Microbial strains and their collection numbers.

Microbial Strain	Collection Number
*Pseudomonas aeruginosa*	B24854
*Acinetobacter baumannii*	B370200
*Proteus vulgaris*	U98727
*Staphylococcus aureus*	B236844
*Citrobacter freundii*	U65813
*Escherichia coli*	U65814
*Enterobacter aerogenes*	B370210
*Candida albicans*	U28732
*Klebsiella pneumoniae*	BA83700

## Data Availability

Not applicable.

## Supplementary Materials

The following supporting information can be downloaded at: https://www.mdpi.com/article/10.3390/molecules27165165/s1, Figure S1: Calibration curves of gallic acid (A), quercetin (B), and tannic acid (C); Table S1. Calibration curves of gallic acid, quercetin, and tannic acid.

## Sample Availability

Samples used in this study are available on request from the corresponding authors.
