# Supplementary material for: Date Fruit (Phoenix dactylifera L.) Cultivar Extracts: Nanoparticle Synthesis, Antimicrobial and Antioxidant Activities
Source: Molecules. 2022 Aug 13;27(16):5165. doi: 10.3390/molecules27165165 (PMC9415038; doi:10.3390/molecules27165165)
Supplement: Supplementary file 1 [file molecules-27-05165-s001.zip › molecules-1850937-supplementary.pdf]

molecules

MDPI

Supplementary Materials

Date Fruit (*Phoenix dactylifera* L.) Cultivar Extracts: Nanoparticle Synthesis, Antimicrobial and Antioxidant Activities

Abdulghani Ashraf Halabi [1], Bassma H. Elwakil [2,*], Mohamed Hagar [3] and Zakia A. Olama [1]

[1] Department of Botany & Microbiology, Faculty of Science, Alexandria University, Alexandria 21568, Egypt
[2] Department of Medical Laboratory Technology, Faculty of Applied Health Sciences Technology, Pharos University in Alexandria, Alexandria 21321, Egypt
[3] Department of Chemistry, Faculty of Science, Alexandria University, Alexandria 21568, Egypt
* Correspondence: bassma.hassan@pua.edu.eg

Table S1. Calibration curve of the gallic acid, quercetin and tannic acid

**Concentration of gallic acid, quercetin or tannic acid** (µg/ml)	**Absorbance of gallic acid**	**Absorbance of quercetin**	**Absorbance of tannic acid**
Blank	0.152 ± 0.02	0.075 ± 0.01	0.250 ± 0.10
0	0.000 ± 0.01	0.000 ± 0.02	0.250 ± 0.09
20	0.170 ± 0.07	0.106 ± 0.06	0.330 ± 0.11
40	0.218 ± 0.01	0.212 ± 0.07	0.420 ± 0.19
60	0.255 ± 0.02	0.340 ± 0.08	0.515 ± 0.21
80	0.305 ± 0.09	0.400 ± 0.11	0.630 ± 0.11
100	0.355 ± 0.04	0.510 ± 0.09	0.725 ± 0.32
200	0.372 ± 0.08	0.600 ± 0.19	0.940 ± 0.39

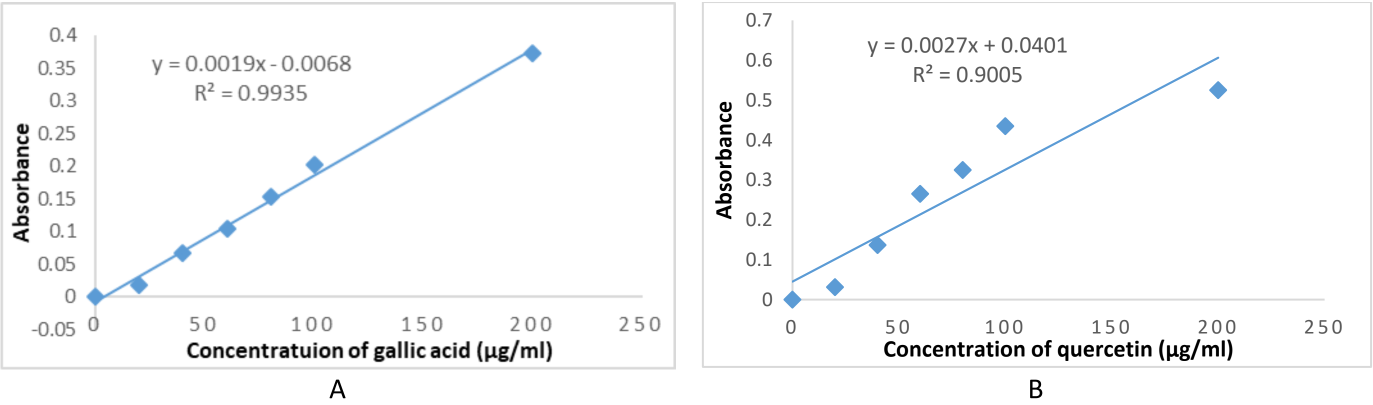

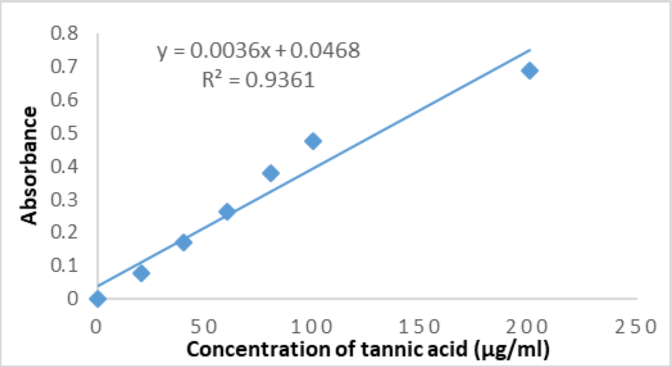

C

Figure S1. Calibration curve of the gallic acid (A), quercetin (B) and tannic acid (C)
